# LILRB3 suppresses immunity in glioma and is associated with poor prognosis

**DOI:** 10.1002/ctm2.1396

**Published:** 2023-10-13

**Authors:** Qiyuan Zhuang, Ying Liu, Hanze Wang, Ziyang Lin, Li Sun, Yue Liu, Yingying Lyu, Liang Chen, Hui Yang, Ying Mao

**Affiliations:** ^1^ Department of Neurosurgery Huashan Hospital Fudan University Shanghai P. R. China; ^2^ Department of Pathology, School of Basic Medical Sciences Fudan University Shanghai P. R. China; ^3^ Institute for Translational Brain Research, Shanghai Medical College Fudan University Shanghai P. R. China; ^4^ National Center for Neurological Disorders Huashan Hospital Shanghai Medical College Fudan University Shanghai P. R. China; ^5^ Shanghai Key Laboratory of Brain Function Restoration and Neural Regeneration, Shanghai Clinical Medical Center of Neurosurgery, Neurosurgical Institute of Fudan University, Huashan Hospital, Shanghai Medical College Fudan University Shanghai P. R. China; ^6^ State Key Laboratory of Medical Neurobiology and MOE Frontiers Center for Brain Science, Shanghai Medical College Fudan University Shanghai P. R. China

Dear Editors,

Gliomas are the most prevalent type of primary brain tumour. Within the glioma tumour microenvironments (TMEs), tumour‐associated macrophages (TAMs) that hold high expression of co‐inhibitory receptors constitute a significant portion of the immune landscape.^1^, ^2^ The effects analysis of co‐stimulatory/inhibitory receptors mRNA levels on overall survival and multivariable Cox regression identified LILRB3 as an independent negative prognostic factor among various grades in glioma (Figures S1A–C). In the Huashan glioblastoma (GBM) cohort, we observed that patients with dense LILRB3 had significantly worse survival outcomes (*n* = 78, Figure 1A,B), independent of treatment strategy and tumour location (Figure S1D). These findings were validated in the CGGA_325 cohort, CGGA_693 cohort, GSE83300 and GSE68848 (Figure S1E–H). Single‐cell RNA‐sequencing (scRNA‐seq) of TAMs from GBM patients^3^ showed LILRB3 was predominantly expressed on monocyte‐derived TAMs rather than microglia‐derived TAMs (Mg‐TAMs) (Figure 1C). We found a significant colocalization of CD206 and LILRB3 in the cell membrane (Figure 1D), and increased infiltration of LILRB3^+^ CD206^+^ cells in the tumour region (Figure 1E). The detailed analysis or experiment methods are described in the Supporting Information.

**FIGURE 1:**
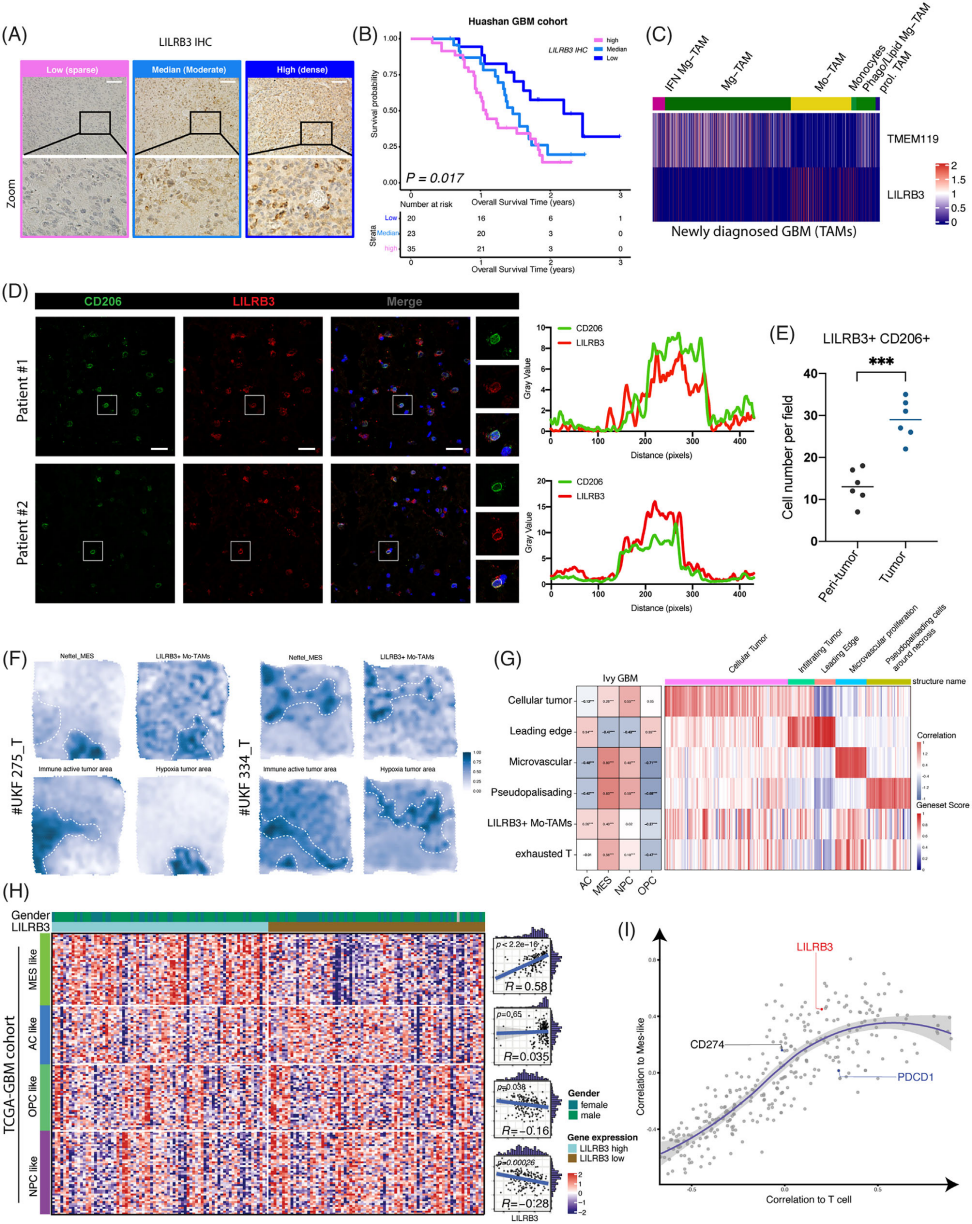
High LILRB3 localized in monocyte‐derived tumour‐associated macrophages (MDMs) was an unfavourable prognostic factor in glioma. (A) Immunohistochemistry staining of LILRB3 in Huashan glioblastoma (GBM) cohort (*n* = 78). Shown are representative images from patients with similar results. The immunohistochemistry (IHC) staining score of each sample was evaluated by staining intensity, and divided into three groups (dense, moderate, and sparse). Scale bar: 100 μm. (B) Kaplan‐Meier analysis of correlations between LILRB3 IHC levels and the overall survival of patients with GBM in the Huashan cohort. The *p*‐value was from Kaplan–Meier log‐rank test. (C) Scaled heatmap of microglia marker TMEM119 and LILRB3 expression from scRNA‐seq data. (D) Left, Immunofluorescence staining with LILRB3 and CD206 in two GBM cases. Scale bar: 20 μm. Right, Quantification of immunofluorescence (IF) signal of LILRB3 and CD206 in the zoom area of two cases. (E) LILRB3^+^ CD206^+^ cells were counted in tumour and peri‐tumour regions. (F) Surface signature plots of the integrated analysis in samples #UKF275_T and #UKF334_T. For each case, the MES‐like feature reported by Neftel et al.^6^ (at the top, left), and the LILRB3+ Mo‐TAM is indicated at the top (right). At the bottom, immune active tumour area (left) and hypoxia tumour area (right) are presented. Higher scores are indicated in blue. (G) Heatmap of the correlation and expression of different regions described in Ivy GAP cohort. Left, Pearson's coefficient analysis of groups. Right, expression pattern among different groups. (H) Heatmap and correlation plot of LILRB3 expression with MES‐like, AC‐like, OPC‐like and NPC‐like in TCGA and CGGA databases respectively. Left, heatmap of subtype score among different LILRB3 expressed groups. Right, scatterplot and linear regression of subtype scores and LILRB3. (I) Plot of 320 genes of T cell‐specific and several co‐stimulating or co‐inhibitory. Each gene's expression was calculated to estimate the correlation with T cells, defined by genes (CD2, CD3D, CD3E and CD3G) (X‐axis). The Y‐axis showed the correlation of each gene's expression to the MES‐like scores. The line indicates a LOESS regression, and the grey area showed the confidential region. Colors distinguish marker genes. *p < .05, **p < .01, ***p < .001, ****p < .0001.

The reciprocal interactions between GBM cellular states and TAMs modulate the tumour immune environment.^4^ By spatial 10x transcriptome previously reported by Ravi et al.,^5^ we found that LILRB3^+^ Mo‐TAMs had close proximity to mesenchymal (MES‐like) states, rather than other cellular states (Figure 1F,H). Besides, LILRB3^+^ Mo‐TAMs, highly expressed in the microvascular region, are closely associated with exhausted T cells and MES‐like features in the Ivy GAP cohort (Figure 1G). Other cellular signatures proposed by Neftel et al.^6^ exhibited negative or no correlation with LILRB3 expression. Higher correlation coefficients of LILRB3 with MES‐like scores were shown by locally weighted regression (Figure 1H,I and Figure S2A,B). Macrophages cocultured with MES28 cells (MES‐like glioma stem cells) performed a significantly higher mean LILRB3 MFI level (Figure 2C–E) and higher gene expression (Figure 2F). Since high MES‐like expression was associated with a worse prognosis in both IDH mutant and IDH wild‐type glioma patients (Figure S3A,B), we found most of the mesenchymal genes were upregulated in temozolomide (TMZ)‐resistant U251. Moreover, concurrent TMZ treatment increased the infiltration percentage of IFNγ^+^ CD4^+^ and IFNγ^+^ CD8^+^ in the GL261 mouse model and upregulated mesenchymal gene expression (Figure S3C,E–M). These findings are consistent with the higher LILRB3 expression of recurrent GBM (Figure 2A,B) in the GLASS cohort (primary and recurrent paired samples, *n* = 86) and in TAM clusters of both primary and recurrent samples.^3^ Notably, 19q13.42, where the LILRB3 is located, was the most frequently deleted locus in TCGA‐LGG (54%) (Figure S2E,F). LILRB3 expression was lower in the 1p/19q codeletion group (Figure S2C,D,H, I), and significantly affected by the deletion status of 19q13.42 by marker‐based scores analysis (Figure S2E).

**FIGURE 2:**
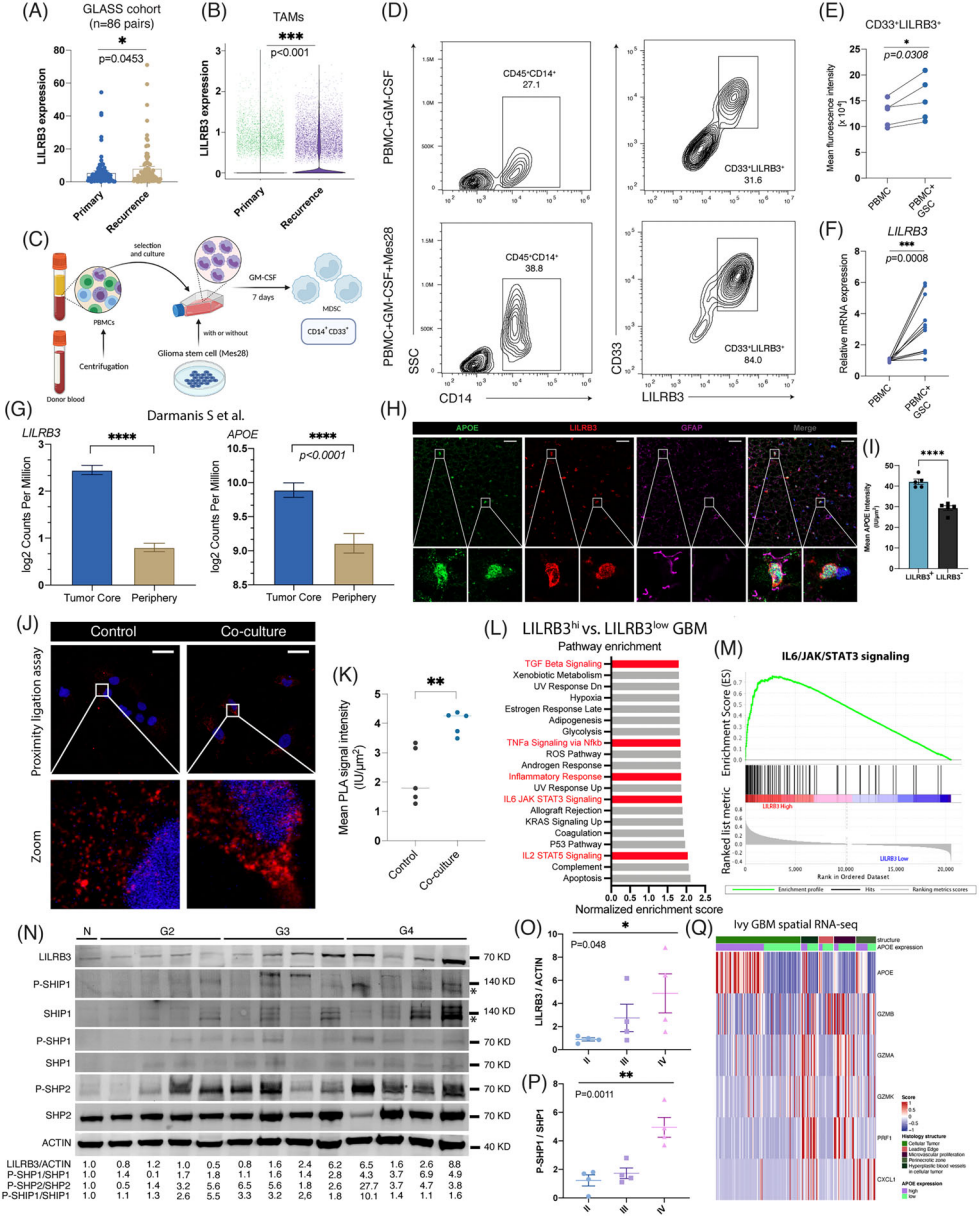
Expression of LILRB3 binding with Apolipoprotein E (APOE) was positively correlated with the mesenchymal‐like cellular state in glioblastoma (GBM) and upregulated by MES‐like subtype of GBM cells. (A) LILRB3 expression was analyzed among paired primary and recurrent GBM in the glioma longitudinal analysis (GLASS) cohort. The patients are filtered based on whether they received concurrent treatment with temozolomide (TMZ). (B) LILRB3 expression in tumour‐associated macrophages (TAMs) clusters derived from primary and recurrent GBM (C) Schematic diagram of PBMC and MES‐like glioma coculture system. (D) The expression of LILRB3 and other proteins was examined by flow cytometry after 7 days of coculture. (E) Quantification of CD33+LILRB3+ expression from harvested cells among two groups. Data are shown as dots (*n* = 5). (F) Relative expression of LILRB3 from harvested cells among two groups. (G) Quantification of LILRB3 and APOE expression in Darmanis et al. data set among tumour and peripheral region.^9^ (H) IF staining of APOE, LILRB3 and GFAP. Scale bar: 20 μm. (I) Quantification of mean APOE intensity around LILRB3^+^ and LILRB3^−^ cells. (J) Proximity ligation assay of APOE and LILRB3 with or without tumour cell medium in HMC3 cells. Cells are harvested after 24 h co‐culture. The red dot implicated the binding signal of two proteins. Scale bar: 20 μm. (K) Quantification of proximity ligation assay (PLA) signals among control and co‐culture groups. Five repeated cases are shown. (L) Gene Set Enrichment Analysis (GSEA) of high LILRB3 versus low LILRB3 TCGA‐GBM. Significant pathways were ranked by normalized enrichment score. Red means potential pathway contributed to tumour progression (M) GSEA of indicated HALLMARK pathway of high LILRB3 TCGA‐GBM. (N) Immunoblotting of different grades of glioma showed that LILRB3 was elevated along with grades, compared to non‐tumour samples (epilepsy). The density of the blot is quantified at the bottom. Shown are representative blots from at least three separate experiments with similar results. (O, P) Quantification of LILRB3/ACTIN and p‐SHP‐1/SHP‐1 showed these two proteins increased along with grades. Enrichment profile, hits, and ranking metrics scores are plotted. (Q) Expression of APOE and activated CD8 T cell marker genes in different region of GBM. Scores are normalized by the Z score. MES‐like, mesenchymal‐like; NPC‐like, neural progenitor‐like; OPC‐like, oligodendrocyte progenitor‐like; AC‐like, astrocyte‐like; CGGA, Chinese Glioma Genome Atlas.

Apolipoprotein E (APOE) has been reported to be a major lipid carrier, transporting lipids to cells and tissues.^7^ Using 7‐tesla‐field‐strength magnetic resonance spectroscopy, we found Lipid to Creatine ratio in high‐grade gliomas is around 1.30 (Figure S2J), higher than the ratio in normal brain tissue around 0.59.^8^ Notably, LILRB3 and APOE were expressed higher in the central region (Figure 2G).^9^ Abundant expression of APOE in LILRB3^+^ myeloid cells was observed compared to LILRB3^−^ cells (Figure 2H,I). We then used proximity ligation assay to examine the interaction of APOE and LILRB3 after coculture in situ. Coculture caused increased binding signals in HMC3 compared with the control group (Figure 2J,K). Gene Set Enrichment Analysis of HALLMARK gene sets revealed high enrichment scores for IL6/JAK/STAT3 signaling in the high‐LILRB3‐expressed GBM (Figure 2L,M). Higher LILRB3 and p‐SHP1/SHP1 in high‐grade glioma tissues were observed compared to lower‐grade glioma or non‐neoplastic brain by immunoblotting (Figure 2N–P). This finding is in line with previous reports demonstrating that LILRB3 can interact with SHP‐1 in 293T cells.^10^ Moreover, high expression of APOE shows a lower T cell activation expression pattern in GBM (Figure 2Q).

Notably, a low expression level of PD‐1 and PD‐L1 was detected (Figure S4A–E) in TAMs. ScRNA‐seq and immunofluorescence staining showed almost no co‐labeled PD‐1 and LILRB3 (Figure 3A–C). Based on immunohistochemistry (IHC) staining, only 5% of glioma tissues with low LILRB3 expression exhibited high PD‐1 expression, while both low and high PD‐1 expression was observed in high‐LILRB3‐expression samples (Figure 3D,E and Figure S7A). Although mediating effects analysis showed that TMZ treatment was protective for patients with high LILRB3 expression (indirect effect of 0.73, ratio of 47.3%) (Figure S3D), these findings indicate that high LILRB3 may hold an extra immunosuppressive effect besides PD‐1. Subgroups are divided by LILRB3 and PDCD1 classifier genes in both TCGA and CGGA datasets: double‐high, median (high expression of either LILRB3 or PDCD1), and double‐low (Figure 3F). The optimal cluster number was determined using NMF and k‐means unsupervised clustering (Figures S5 and S6). The double‐high groups had significantly higher Treg and myeloid‐derived suppressor cell signature scores compared to the median or double‐low groups (Figure 3G and Figure S7B–G).

**FIGURE 3:**
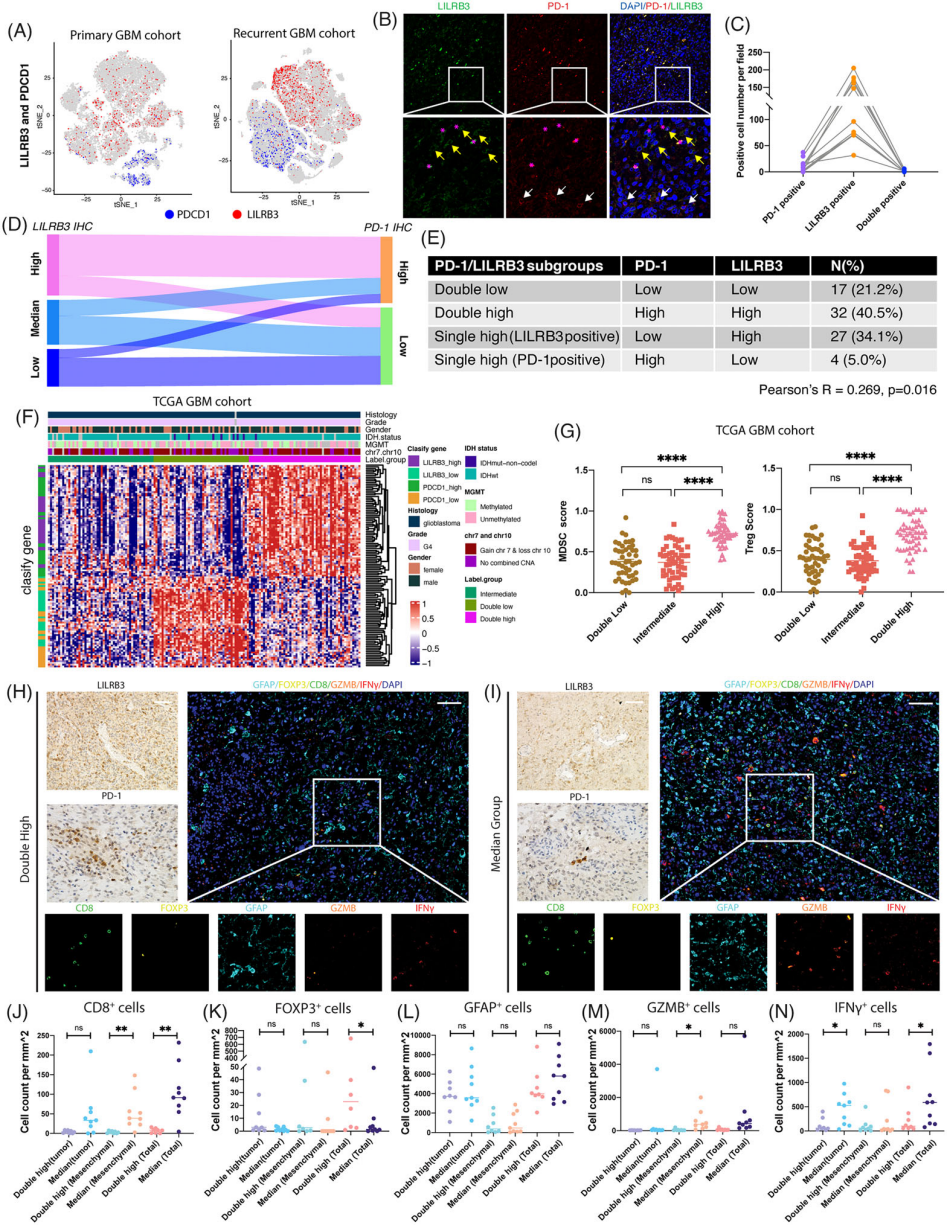
LILRB3 and PD‐1 respectively distributed in myeloid cells and T cells contributing to the immunosuppressive microenvironment in glioma. (A) The distribution of LILRB3 and PDCD1 in t‐SNE reduction plot of single‐cell RNA sequencing data (10x genomics) from enriched CD45^+^ cells in newly diagnosed glioblastoma (GBM) and recurrent GBM. (B) Double immunofluorescence staining with LILRB3 and PDCD1 (PD‐1) revealed respective distribution patterns in patients. Shown are representative images from at least five cases with similar results. Images of higher magnification are shown at the bottom. Representative LILRB3 or PD‐1 positive cells were marked with an arrow (yellow) or arrow (white), a pink star denotes nonspecific signals. Scale bar: 20 μm. (C) Positive cell number per field in captured images was counted (case number: *n* = 8). (D) Sankey plot of relationships between LILRB3 staining score with PDCD1 immunohistochemistry (IHC) score. (E) Chi‐square analysis of samples annotated with distinct subgroups. Samples with high expression or median expression of LILRB3 are classified into high LILRB3 group for Chi‐square analysis. (F) Heatmap of the unsupervised cluster of expression of the classified gene in TCGA GBM dataset, respectively. Scores are normalized by the Z score. (G) Myeloid‐derived suppressor cells (MDSC) and Treg enrichment scores across different modules. (H, I) Top left, immunohistochemistry staining of LILRB3 and PD‐1 of samples in groups labeled with double high or median. Shown are representative images from at least five patients with similar results. Top right, multiplex immunohistochemistry staining of samples. Images of higher magnification are shown at the bottom. Scale bar: 100 μm. (J–N) Statistical results of CD8^+^, FOXP3^+^, GFAP^+^, GZMB^+^ and IFNγ^+^ cells across double high and median high groups (number of double high: 8, number of median high: 9). The statistical result was analyzed by ANOVA with post‐t‐test. Mean and SEM. Data are shown as dots. ns, no significance; *p < .05, **p < .01, ***p < .001, ****p < .0001.

We then employed multiplex IHC to quantify FOXP3^+^, IFN‐γ^+^, and GZMB^+^ T cells in the median (*n* = 9) and double‐high (*n* = 8) groups of GBM patients (Figures 3H, I). GFAP^+^ cells did not differ significantly between the two groups, indicating similar absolute numbers of tumour cells (Figure 3L). Compared to the median group, the double‐high group exhibited an increased proportion of FOXP3^+^ T cells and decreased proportions of IFN‐γ^+^ and GZMB^+^ T cells in GBM samples (Figure 3J–N).

In conclusion, we identified LILRB3, which is highly expressed in monocyte‐derived TAMs, as an independent negative prognostic factor among various grades of gliomas. LILRB3 expression was positively correlated with the mesenchymal‐like cellular state in GBM and upregulated after cocultured with MES‐like glioma cells. In glioma TME, LILRB3 was bound with APOE and was accordant with the activation of SHP‐1. PD‐1 and LILRB3 exhibited consistent but different distribution patterns which could be used to identify three glioma immune subgroups. Double‐high group (LILRB3 high and PD‐1 high) exhibited a ‘colder’ TME compared with other groups, indicating LILRB3 as a potential target of PD‐1 antagonist combination therapies.

## CONFLICT OF INTEREST STATEMENT

The authors declare no conflict of interest.

## Supporting information

Supporting Information

Supporting Information

Supporting Information

Supporting Information

Supporting Information

Supporting Information

Supporting Information

Supplementary Table1. Clinical Characteristics of patients in Huashan GBM cohort.

Supplementary Table2. Classify genes for PDCD1 and LILRB3.

Supplementary Table3. Markers genes of activated and immune inhibitory T cell states.

Supporting Information
