# Supplementary figures and images for: LILRB3 suppresses immunity in glioma and is associated with poor prognosis
Source: Clin Transl Med. 2023 Oct 13;13(10):e1396. doi: 10.1002/ctm2.1396 (PMC10570768; doi:10.1002/ctm2.1396)

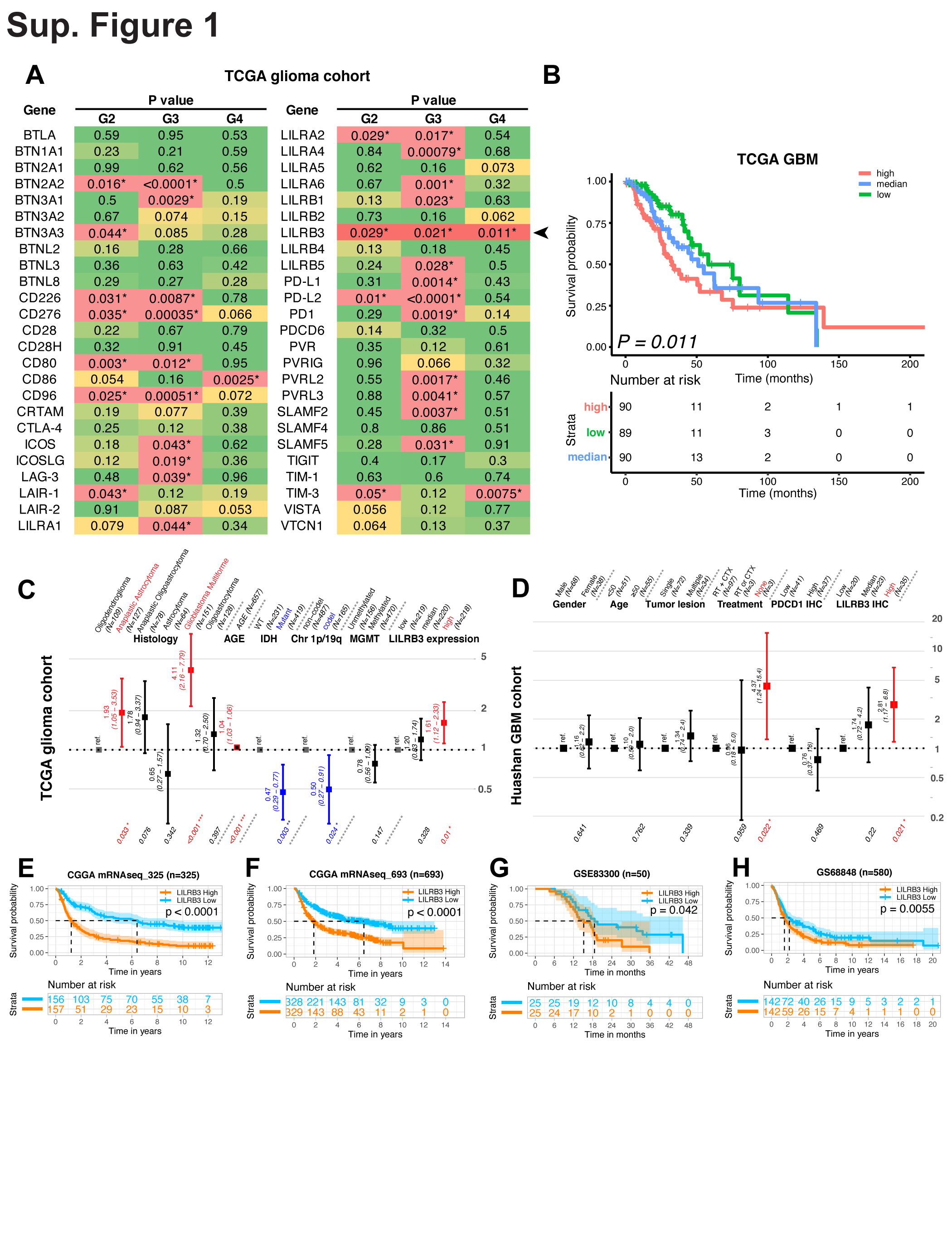

Supplement: Supplementary file 1 — Supporting Information [file CTM2-13-e1396-s001.tif]

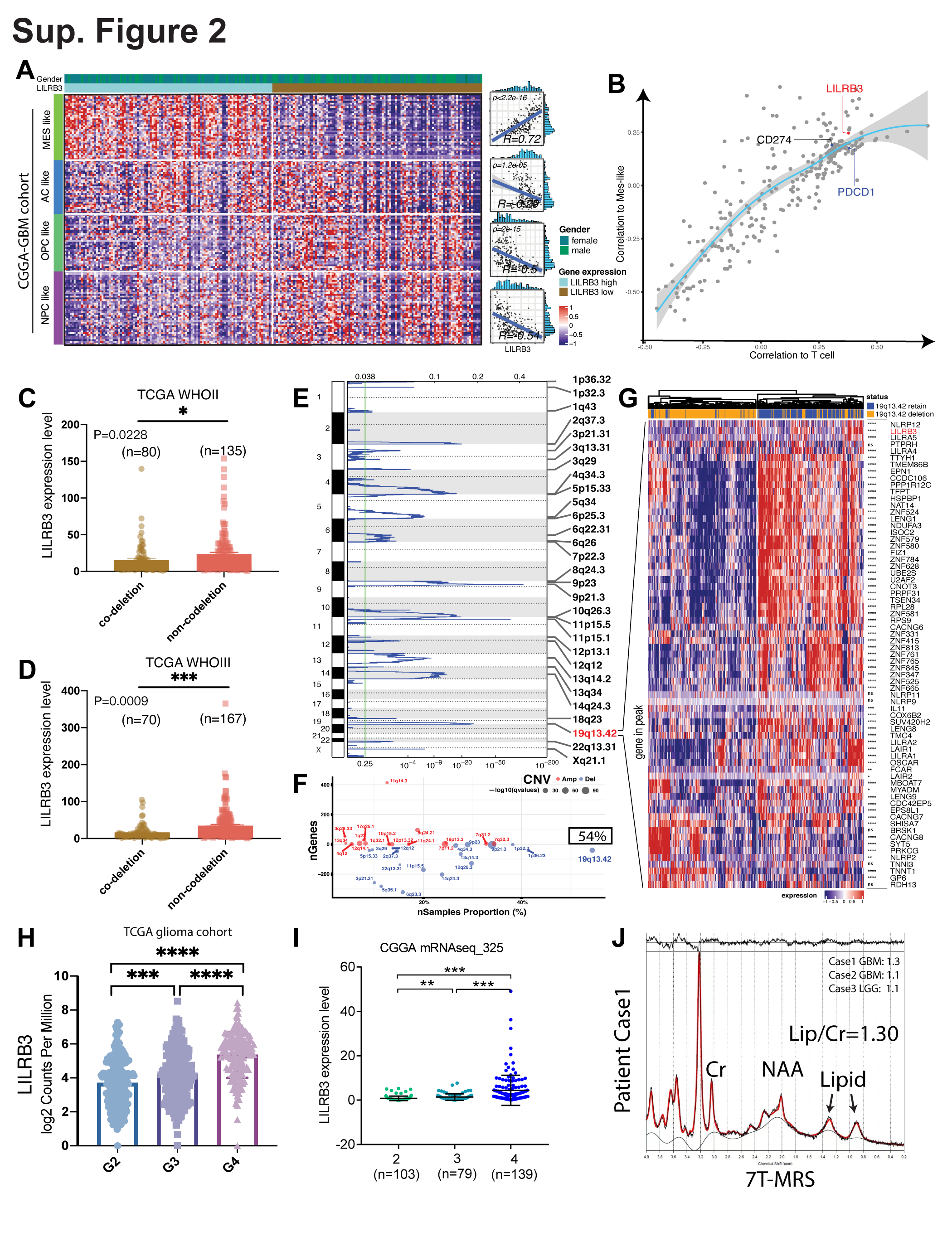

Supplement: Supplementary file 2 — Supporting Information [file CTM2-13-e1396-s002.tif]

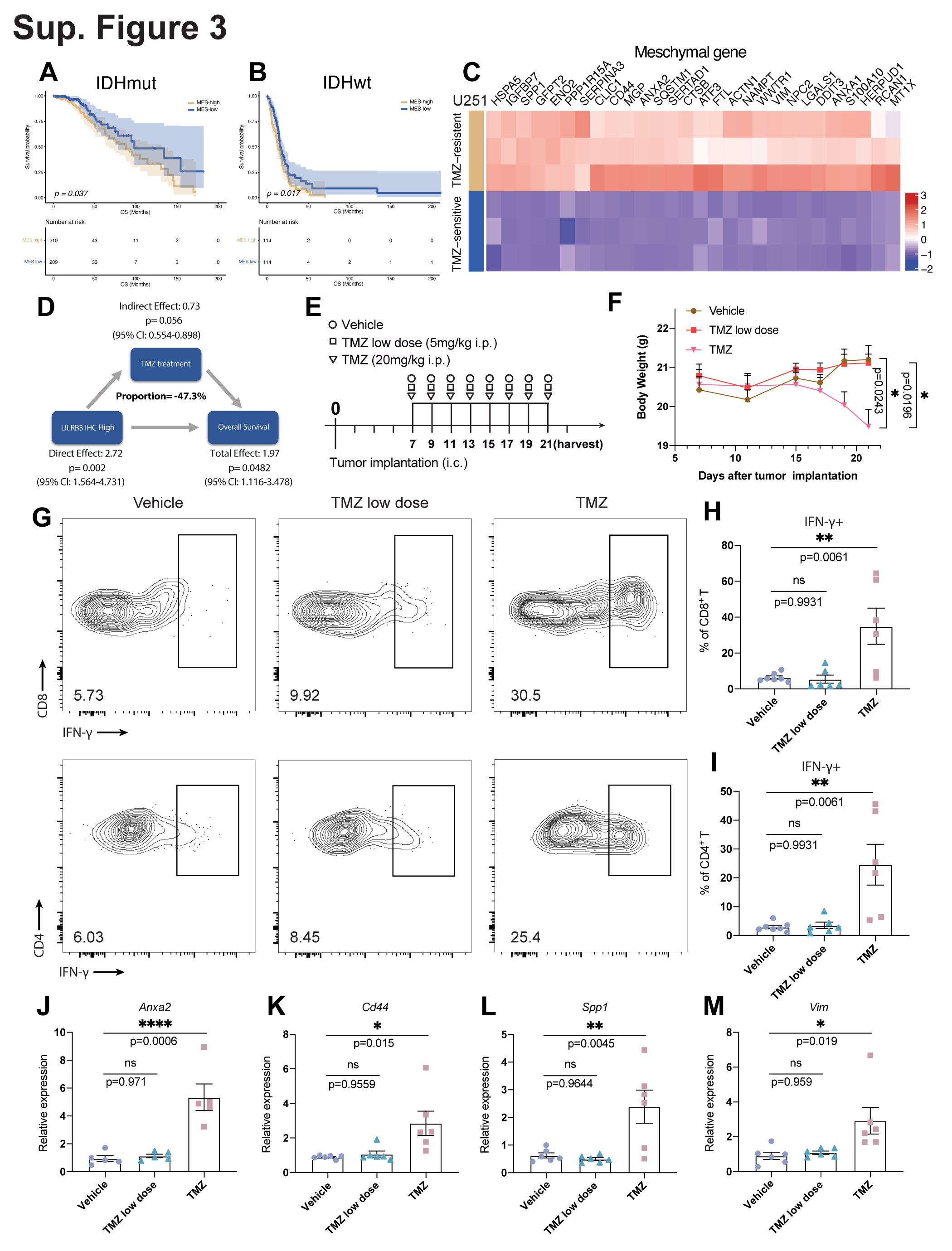

Supplement: Supplementary file 3 — Supporting Information [file CTM2-13-e1396-s010.tif]

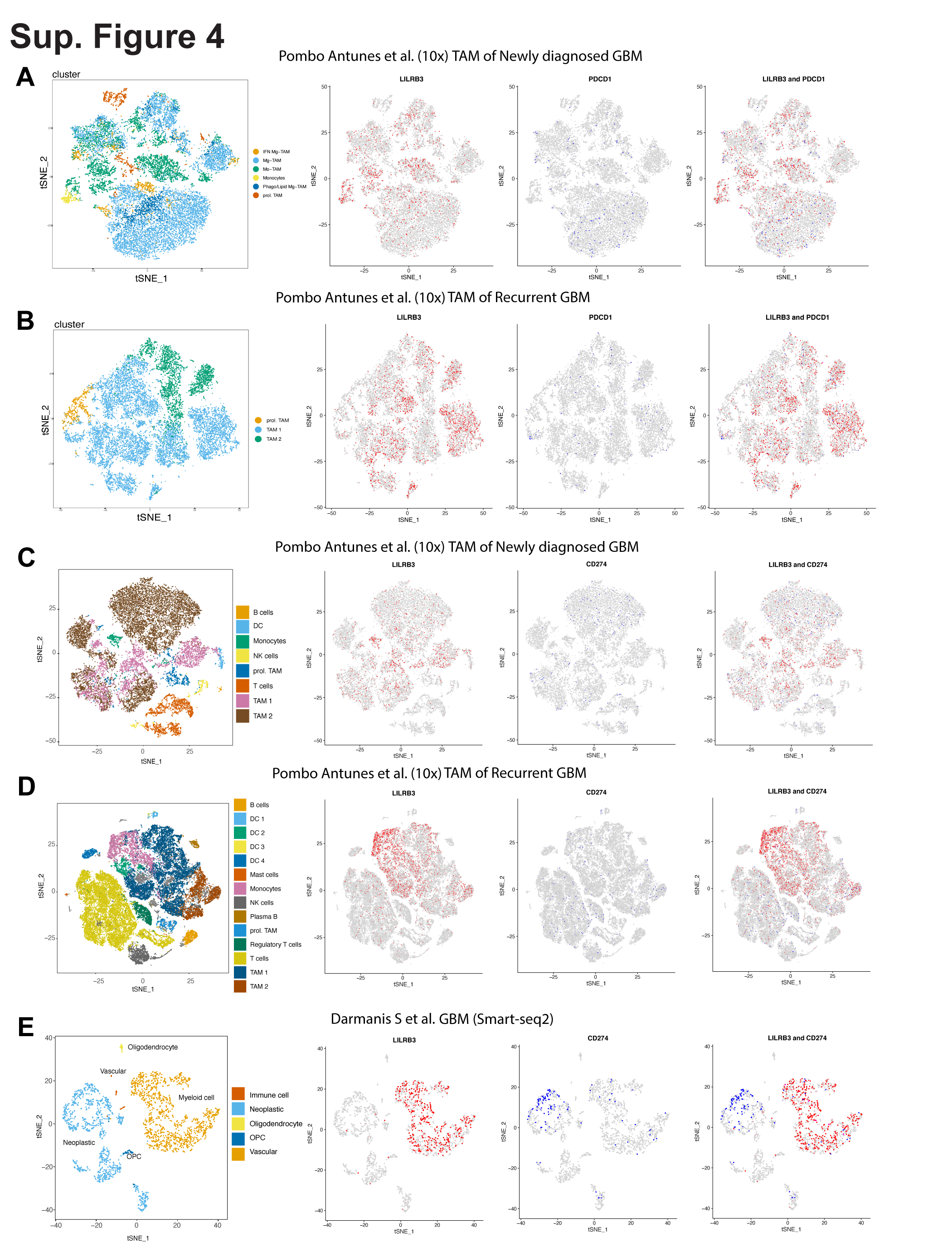

Supplement: Supplementary file 4 — Supporting Information [file CTM2-13-e1396-s003.tif]

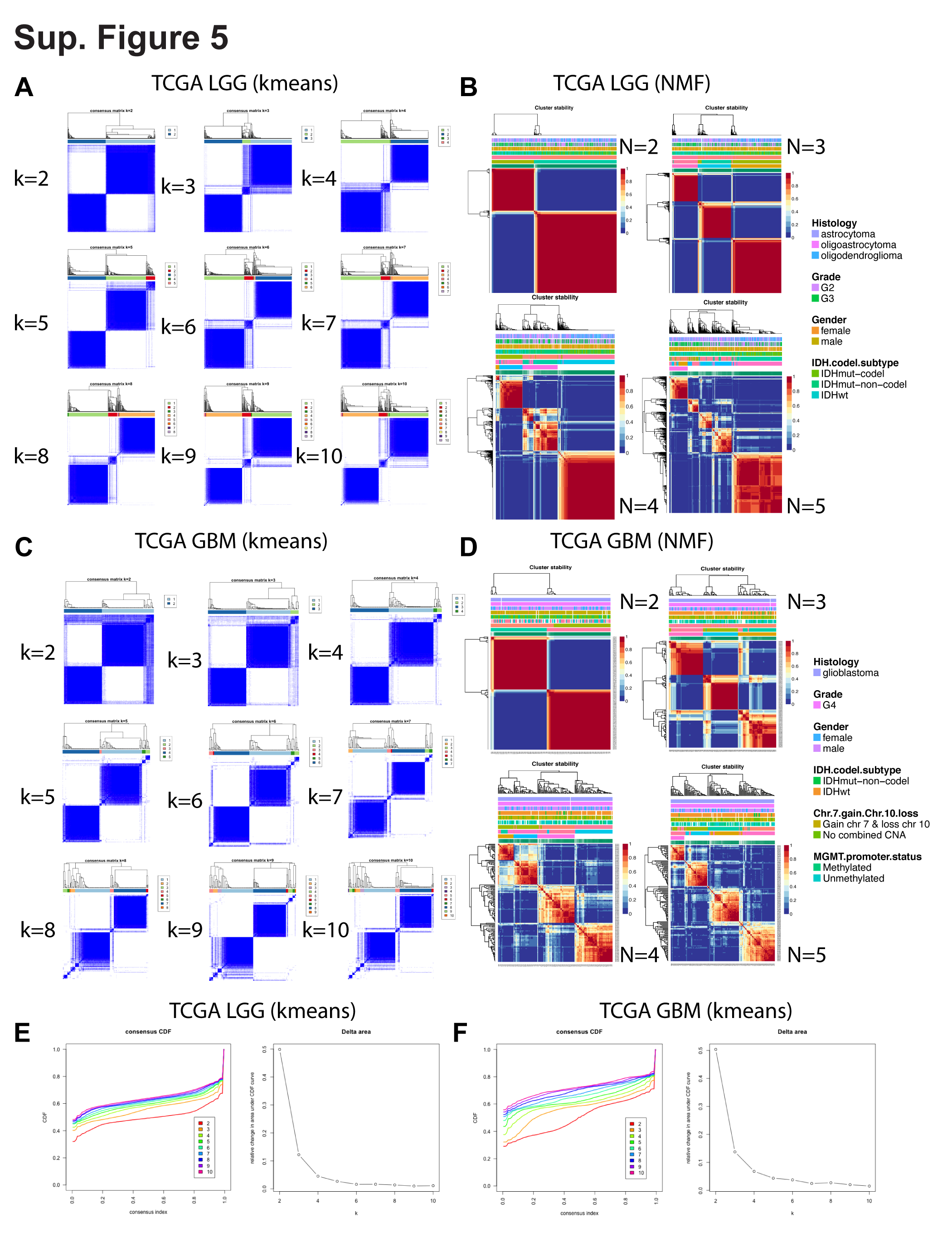

Supplement: Supplementary file 5 — Supporting Information [file CTM2-13-e1396-s005.tif]

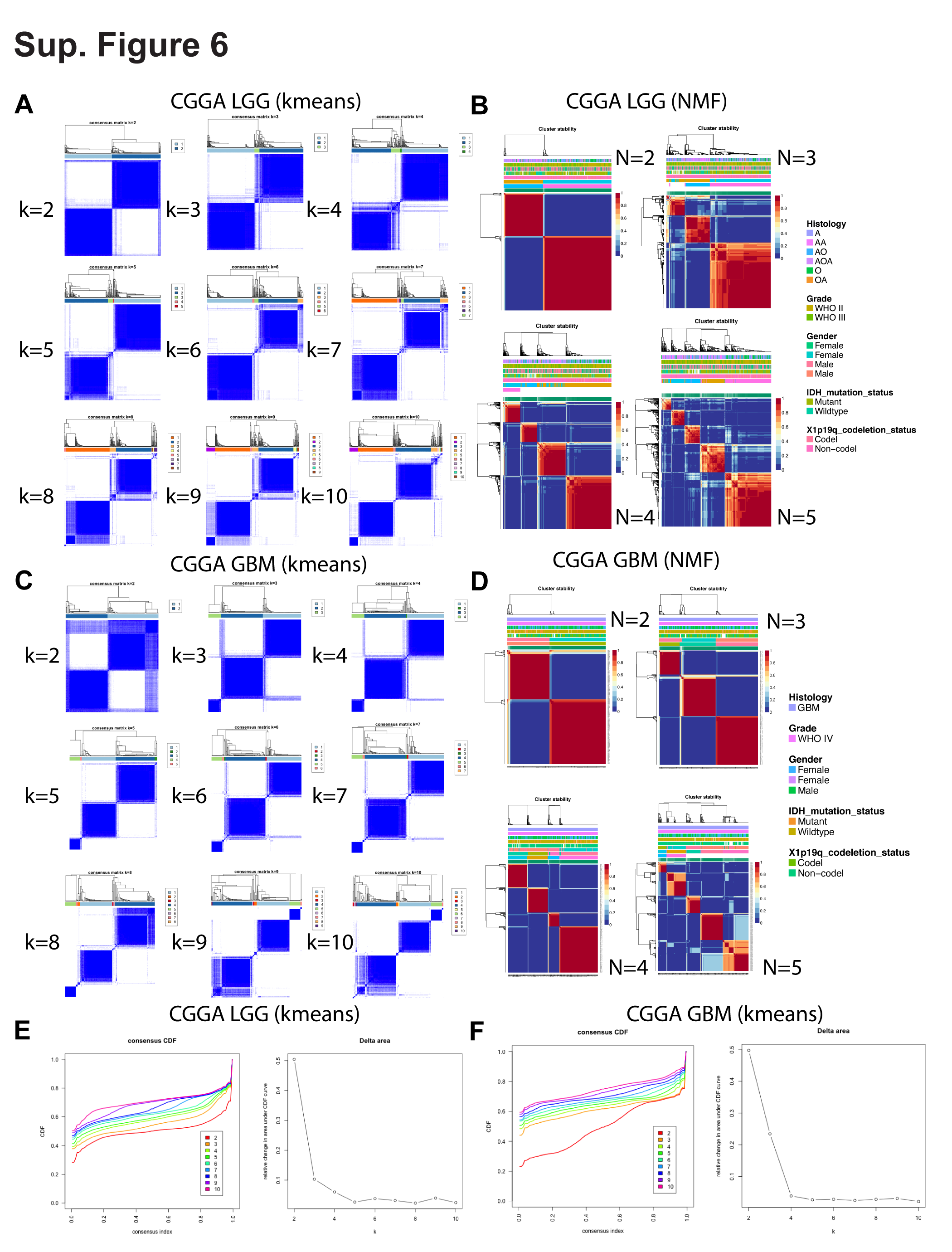

Supplement: Supplementary file 6 — Supporting Information [file CTM2-13-e1396-s006.tif]

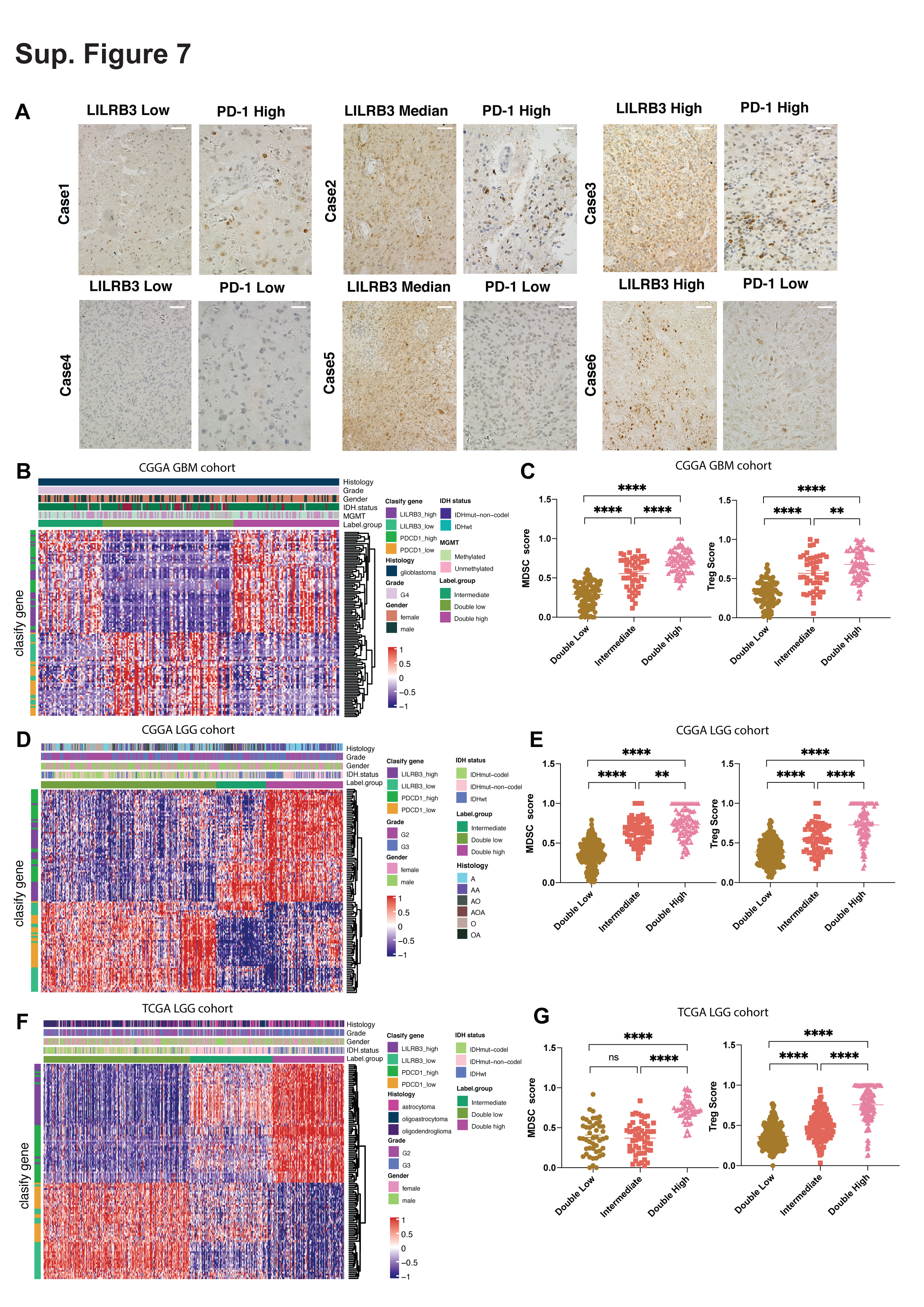

Supplement: Supplementary file 7 — Supporting Information [file CTM2-13-e1396-s004.tif]
