# Supplementary material for: LILRB3 suppresses immunity in glioma and is associated with poor prognosis
Source: Clin Transl Med. 2023 Oct 13;13(10):e1396. doi: 10.1002/ctm2.1396 (PMC10570768; doi:10.1002/ctm2.1396)

**LILRB3 suppresses immunity in glioma tumors and is associated with poor prognosis**

Qiyuan Zhuang^1,7^, Ying Liu^2,7^, Hanze Wang^3,7^, Ziyang Lin^1,7^, Li Sun^1^, Yue Liu^1^, Yingying Lyu^1^, Liang Chen^1,4,5,6^*, Hui Yang^1,3,4,5,6^*, Ying Mao^1,4,5,6^*

^1^ Department of Neurosurgery, Huashan Hospital, Fudan University, Shanghai, P.R. China;

^2^ Department of Pathology, School of Basic Medical Sciences, Fudan University, Shanghai, China

^3^ Institute for Translational Brain Research, Shanghai Medical College, Fudan University, Shanghai, P.R. China;

^4^ National Center for Neurological Disorders, Huashan Hospital, Shanghai Medical College, Fudan University, Shanghai, P.R. China;

^5^ Shanghai Key Laboratory of Brain Function Restoration and Neural Regeneration, Shanghai Clinical Medical Center of Neurosurgery, Neurosurgical Institute of Fudan University, Huashan Hospital, Shanghai Medical College, Fudan University, Shanghai, P.R. China;

^6^ State Key Laboratory of Medical Neurobiology and MOE Frontiers Center for Brain Science and MOE Frontiers Center for Brain Science, Shanghai Medical College, Fudan University, Shanghai, P.R. China;

^7^ These authors contributed equally to this work.

*Correspondence:

Ying Mao, Ph.D., M.D. (E-mail: maoying@fudan.com)

Department of Neurosurgery, Huashan Hospital, Fudan University, 958 Jing’guang Road, Neurosurgical Research Institute, Shanghai 200032, P.R. China

Or Hui Yang, Ph.D. (E-mail: [hui_yang@](mailto:hjwu@shmu.edu.cn)fudan.edu.cn)

Department of Neurosurgery, Huashan Hospital, Fudan University, 131 Dong’an Road, Room 801 Mingdao Building, Shanghai 200032, P.R. China

Or Liang Chen, Ph.D. (E-mail: chenlianghs@126.com)

Department of Neurosurgery, Huashan Hospital, Fudan University, 131 Dong’an Road, Room 801 Mingdao Building, Shanghai 200032, P.R. China

**This file includes:**

Supplementary Figures and Figure Legends (Sup-Figure 1 ~ Sup-Figure 7)

Supplementary Materials and Methods

Supplementary Discussion

Supplementary Reference

**SUPPLEMENTARY FIGURES AND** **FIGURE LEGENDS**

**Supplementary Figure1. High expression of LILRB3 was an independent negative prognostic factor in glioma.** (A) Kaplan–Meier log-rank test results of correlations between the mRNAs of co-inhibitory, co-stimulating receptors and the overall survival of patients with glioma (G2: n=256, G3: n=269, G4:n=164, divided into three groups depending on gene expression) in TCGA database. (B) Kaplan-Meier plot of correlations between LILRB3 mRNA level and the overall survival of patients with GBM in TCGA database. The P-value was from Kaplan–Meier log-rank test. (C) Multivariable COX analysis of clinical characteristics and LILRB3 expression in TCGA glioma cohort (n=689). Red means significant risk factors for overall survival, and blue means protective factors. Hazard ratio, confidential interval, and p-value are listed within the forest plot. (D) Multivariable COX analysis of clinical characteristics and LILRB3 IHC score in Huashan GBM cohort (n=106). Red means significant risk factors for overall survival. (E-H) Kaplan-Meier analysis of correlations between LILRB3 mRNA level and the overall survival of patients with GBM in several glioma databases (E, CGGA mRNAseq_325 cohorts. F, CGGA mRNAseq_693 cohorts. G, GSE83300. H, GSE68848). The P-value was from Kaplan–Meier log-rank test.

**Supplementary Figure2. The expression of LILRB3 was affected by chromosome 1p/19q co-deletion status and associated with mesenchymal-like state in GBM.** (A) Heatmap and correlation plot of LILRB3 expression with MES-like, AC-like, OPC-like and NPC-like in CGGA-GBM database respectively. Left, heatmap of subtype score among different LILRB3 expressed groups. Right, scatterplot and linear regression of subtype scores and LILRB3. (B) Scatterplot of 320 T cell-specific and several co-stimulating or co-inhibitory genes. Each gene’s expression was calculated to estimate the correlation with T cells, defined by genes (CD2, CD3D, CD3E, CD3G) (x axis). Y axis showed the correlation of each gene’s expression to the MES-like scores. The line indicates a LOESS regression, and the grey area showed the confidential region. Colors distinguish marker genes. (C-D)Expression of LILRB3 in 1p/19q codeletion samples or non-codeletion samples in TCGA datasets among different grades. (E) Genomic positions of deleted regions in lower-grade glioma by GISTIC2.0: Normalized deletion signals (X-axis, top), the significance by Q value (bottom), cutoff at Q value=0.25 (Green line). (F) Scatter plot of deleted regions: number of genes in peaks (Y-axis), frequency of sample with deleted regions among all (X-axis, percentage). Red circle, amplification regions; blue circle, deleted regions; circle size q value of several regions. (G) Heatmap of expressions of genes located in 19q13.42 regions across glioma cohort by unsupervised clustering. (H-I) Expression of LILRB3 among each grade of glioma in TCGA and CGGA. (J) Brain 7-T MR spectroscopy of glioma of three glioma patients to evaluate tumor metabolic activity, showing lipid peaks in the tumor part.

**Supplementary Figure 3. Increased MES-like signature after TMZ treatment was an unfavorable prognostic factor in glioma.**

(A-B) Kaplan-Meier plot of different MES-like signature groups in IDH-mut or IDH-wt glioma of TCGA cohort, respectively. The P-value was from Kaplan–Meier log-rank test. (C) MES-like signature expression in TMZ sensitive and resistant U251 cell lines. The relative expression of each gene was plotted by heatmap after the Z-score scale. (D) Intermediation analysis of TMZ treatment on LILRB3 high expressed group based on COX model^1^. Proportion is calculated by log(Indeirct effect)/(Direct effect). (E) Diagram of different TMZ treatment strategies. (F) Body weight change of different TMZ treatment groups after tumor implantation. The P-value was from adjusted examination after Two-way ANOVA. (G) Flow cytometric analysis of IFNγ+ expression on CD4^+^ T and CD8^+^ T subsets. (H-I) Statistical result of the percentage of IFNγ+ CD4^+^ T and IFNγ+ CD8^+^ T among different groups. (J-M) Relative expression of *Anxa2*, *Cd44*, *Spp1* and *Vim* in sorted tumor cells among different groups. ns, no significance; **P* <0.05, ***P* <0.01, *****P* <0.0001. TMZ, tolazamide; MES-like, mesenchymal-like; TCGA, The Cancer Genome Atlas.

**Supplementary Figure4. LILRB3 are major ITIMs compared to PD-1 and PD-L1 in cell clusters.**

(A-B) Left, t-SNE reduction plot of single-cell RNA sequencing data (10x genomics) from enriched CD45+ cells in newly diagnosed GBM and recurrent GBM. Right, features of LILRB3 and PDCD1 expression in t-SNE reduction plot of newly diagnosed GBM and recurrent GBM.

(C-D) Left, t-SNE reduction plot of single-cell RNA sequencing data (10x genomics) from enriched CD45+ cells in newly diagnosed GBM and recurrent GBM. Right, features of LILRB3 and PD-L1 (CD274) in t-SNE reduction plot. (E) Left, t-SNE reduction plot of single-cell RNA sequencing data (Smart-seq2) from GBM. Right, features of LILRB3 and PD-L1 (CD274) in t-SNE reduction plot.

**Supplementary Figure 5. TCGA cohort optimal cluster number identification based on LILRB3 and PDCD1 classify gene expression.** (A) k-means non-supervised clustering (k number set from 2-10). (B) NMF clustering (N number set from 2-4). (C) k-means non-supervised clustering (k number set from 2-10). (D) NMF clustering (N number set from 2-4). (E-F) left, plot of CDF value of each k number in TCGA LGG and GBM cohorts; right, delta area of CDF curve among each k number in TCGA LGG and GBM cohorts.

**Supplementary Figure 6. CGGA cohort optimal cluster number identification based on LILRB3 and PDCD1 classify gene expression.** (A) k-means non-supervised clustering (k number set from 2-10). (B) NMF clustering (N number set from 2-4). (C) k-means non-supervised clustering (k number set from 2-10). (D) NMF clustering (N number set from 2-4). (E-F) left, plot of CDF value of each k number in CGGA LGG and GBM cohorts; right, delta area of CDF curve among each k number in CGGA LGG and GBM cohorts.

**Supplementary Figure 7. LILRB3 and PD-1 double-high group showed an immunosuppressive microenvironment in GBM.** (A) Immunohistochemistry staining of LILRB3 and PD-1 in Huashan GBM cohort (n=78). Shown are representative images from patients with similar results. The IHC staining score of each sample was evaluated by staining intensity. Scale bar: 100μm. (B, D, F) Heatmap of the unsupervised cluster of expression of the classified gene in TCGA-LGG, CGGA-LGG and CGGA-GBM dataset, respectively. Scores are normalized by the Z score. (C, E, G) MDSC and Treg enrichment score across different modules. The P-value was from ANOVA and post-t-test. **P* <0.05, ***P* <0.01, ****P* <0.001, *****P* <0.0001.

**SUPPLEMENTARY MATERIALS AND METHODS**

**Patients and samples**

After obtaining approval of the institutional review board, we conducted a retrospective study of patients who performed GBM section surgery at the Department of Neurosurgery, Huashan Hospital affiliated to Fudan University (114 cases, named Huashan GBM cohort, the detailed ID and clinical characteristics are listed in Table S1) from January 2010 to August 2011. Informed consent was obtained from each patient in cohorts. Formalin-fixed paraffin-embedded tissues were available from cases, and HE staining of tumor slides was used to confirm the grade. The diagnosis was made by two senior pathologists according to the glioma classification criteria of WHO 2021. Overall survival (OS) and progression-free survival (PFS) were calculated from tumor resection to death or the last follow-up, which was December 31, 2013.

**Transcriptome data collection and analysis**

RNA-seq expression profiles and clinical characteristics of the cancer genome atlas (TCGA) glioma cohort, Chinese Glioma Genome Atas (CGGA) and The Glioma Longitudinal AnalySiS (GLASS) were downloaded. Glioma with expression profiling by array obtained from the Gene Expression Omnibus (GEO, http://www.ncbi.nlm.nih.gov/geo/) database, accession number GSE83300^2^ and GSE68848^3^. RNA sequencing expression data were further normalized using FPKM, and mRNAs were quantified using GRCh38 as the reference genome. Log-rank survival models and COX proportional risk models for each gene or multiple combinations were performed using the survival package in R. Kaplan-Meier survival curves were used for survival visualization. Pearson's correlation test was applied for correlation relationship analysis.

**Identification of PDCD1/LILRB3 classify genes and clustering**

Classifier methods followed previously reported pipelines^4^. In summary, the TCGA-LGG cohort was used for classifying gene discovery. Meanwhile, the TCGA-GBM cohort, CGGA-GBM cohort, and CGGA-LGG cohort were used for validation. Each pair of the gene was examined by multiple biweight midcorrelations and the corresponding Student p-values. The top 50 and the last 50 genes were selected as the positive and the negatively related marker gene, respectively. Since the highly expressing correlation profile between PDCD1 and LILRB3, the intersected gene was excluded. Thus, a total of 80 genes for PDCD1 and 66 genes for LILRB3 were enrolled, which was listed in Table S2. Following this, we performed a non-negative matrix factorization (NMF) algorithm with NMF and CancerSubtypes (R package). we executed 50 runs of NMF and the corresponding random data sets with ranks from 2 to 5. The residual sum of squares (RSS) was compared in each rank, and the consensus matrix of clustering labeled with NMF rank was following generated. Hierarchical clustering performed by ConsensusClusterPlus was also applied for validating the optimized cluster number. Silhouette Similarity for interpretation and validation of consistency within clusters was also derived.

**PBMCs isolation and generation of human myeloid-derived suppressor cells**

Healthy human peripheral blood from five individual donors was collected. Peripheral blood mononuclear cells (PBMCs) were isolated by Ficoll via density gradient centrifugation following the manufacturer’s instructions. To generate a cancer cell cocultured system with M-MDSCs, PBMCs were cultured in RPMI-1640 (Gibco BRL, USA) containing 10% fetal bovine serum (Gibco BRL, USA), 100 mg/mL streptomycin (Gibco BRL, USA) and 20 ng/mL GM-CSF (R&D Systems) at 37°C with or without mesenchymal glioma stem cell (MES3128, 1:50) for 7 days in transwell chamber. For each donor, cultured PBMCs were arranged as the negative control. Medium with fresh cytokines was supplemented on day 4. Cells were harvested following flow cytometry analysis, after 7-days of co-culture. coculture system for HMC3 are similar to PBMC.

**Single-cell and spatial transcriptome data collected and analysis**

The scRNA-seq data were downloaded from two previously reported datasets http://gbmseq.org/ and http://www.brainimmuneatlas.org/. The scRNA-seq data from Darmanis S et al. collected 4 human glioma samples with a total of 3,589 cells, containing 2,343 cells of tumor cores and 1,246 cells of peripheral tumor regions^5^. The tumor cores cells were selected for visualization in this study. Meanwhile, The scRNA-seq data from Pombo Antunes et al. utilized CD45^+^ beads for the enrichment of immune cells from newly diagnosed GBM and recurrent GBM^6^. 21303 cells and 42870 cells with previously annotated were enrolled for analysis. Sequencing settings according to the recommendations of 10× Genomics were loaded on an Illumina HiSeq4000 or Illumina NextSeq 500. We applied the Seurat package in R 4.0.3 for quality control and following data exploration. Low-quality cells were excluded by quality control standards as previously reported. The minimum and maximum cut-off values of features for the plot is set as q5 and q80. The t-distributed stochastic neighbor embedding (t-SNE) is used for visualization. Spatial transcriptome data were downloaded from (https://github.com/theMILOlab/SPATAData), and analyzed following the SPATA guidelines (https://themilolab.github.io/SPATA2/).

**GSEA, ssGSEA analysis**

GSEA software (http://software.broadinstitute.org/gsea/index.jsp) was utilized for the identification of the related pathways of glioma transcriptomic datasets in different expression groups of LILRB3. The false-positive results were corrected by the Benjamini-Hochberg false discovery rate (FDR) method with the adjusted P-value. The statistically significant enriched pathways of the HALLMARK collection derived from MsigDB were plotted and ranked according to the normalized enriched score. Moreover, we used ssGSEA to estimate the gene signature expression profile.

**Definition of glioma signature scores**

Gene signatures of cells were scored according to previously described^7,8^. We derived the validated gene lists from the R package scalop (https://github.com/ jlaffy/scalop). The state score of GBM was following calculated by Ucell (carmonalab/UCell).

**Associations of MES-like state with T-cell and inhibitory states in TCGA**

To evaluate the association between MES-state and subtype of T cells, as well as inhibitory markers. We used the bulk expression profiles of the TCGA-GBM cohort. Specific T cell markers and inhibitory markers were listed in Table S3, being consistent with Figure S1A. Further, we scored each tumor in the TCGA dataset for MES-like state scores and T cell-specific genes scores (CD2, CD3D, CD3E, CD3G). The correlation of genes listed to MES-like state and T-cells were scatter plotted, corresponding to the previous report^7^. Pearson correlation of each gene’s normalized expression to the MES-like score was calculated across all tumors. LOESS regression was applied for examining the confidential interval. LILRB3 and PDCD1 were marked in the plot.

**Immunohistochemistry and immunofluorescence staining**

Human glioma tissues were fixed by 4% paraformaldehyde for FFPE sections (4μm), which followed by staining after deparaffinized and rehydrated. Immunohistochemistry staining procedures were performed as described previously^9^. In brief, 3% H_2_O_2_ was applied for removing endogenous peroxidase, then, the antigen was retrieved by citrate buffer and blocked by 5% normal sheep serum. Primary antibodies were incubated at 4°C overnight (LILRB3 1:100, PDCD 1:50, CD3 1:100; CD8 1:50, GFAP 1:200, APOE 1:100). The secondary antibodies were following incubated for one hour. Liquid DAB+ substrate chromogen system (Dako, USA) and co-stained hematoxylin were used for visualization. As for immunofluorescence (IF) staining, the sections were incubated with Alexa Flour^TM^ 549 goat anti-rabbit IgG (Invitrogen, 2165334) and Alexa Flour^TM^ 488 goat anti-mouse IgG (Invitrogen, 2180683) for 1 h, after primary antibodies incubation and PBS wash. The results were captured using the Leica SP8 system.

**Proximity ligation assay**

The PLA fluorescent experiment follows the Duolink® PLA Product Selection Guide. Primary antibodies of LILR3B and APOE are used to detect the proteins of interest. Incubation, ligation, amplification, and washing are performed according to the manufactory recommendation (Sigma, DUO92101).

**qPCR**

RNA was extracted using the EZ-press RNA purification kit (B0004D, EZBioscience, USA), and quantified using a UV spectrophotometer. RNA was reverse transcribed into cDNA and amplified using an EZ-press Cell to cDNA Kit (B0003, EZBioscience, USA). qPCR was performed on a QuantStudio 6 Real-Time PCR system (Applied Biosystems, Foster City, CA, USA) using SYBR Green qPCR Master Mix (Vazyme Biotech, Nanjing, China). The primers used were as follows: LILRB3 sense strand: 5’-GTCTGGGAAGATACCTGGAGG-3’, antisense strand: 5’-GGACGCTGGAAATCAGTCTTT-3’. All qPCR were repeated three times. The t test was used for comparisons between two normally distributed groups, and one-way analysis of variance was used for comparisons between three or more normally distributed groups. P < 0.05 was considered statistically significant. SPSS 20.0 and GraphPad Prism 8 were used for statistical analysis.

**Antibodies**

Antibodies used in this study included LILRB3 (Abcam, polyclonal, ab271287/Invitrogen, PA5-97926), PD-1 (Abcam, polyclonal, ab137132), CD3 (R&D, monoclonal, MAB100), CD8 (R&D, monoclonal, MAB3801), SHP1 (Cell Signaling Technology, 3759S), p-SHP1 (Cell Signaling Technology, 8849S), SHP2 (Cell Signaling Technology, 3397S), p-SHP2 (Cell Signaling Technology, 3703S), SHIP1 (Cell Signaling Technology, 2727S), p-SHIP1 (Cell Signaling Technology, 3941S), LCK (Cell Signaling Technology, 2984S), p-LCK (Invitrogen, PA5-105466)

**Multiplex immunohistochemistry**

We performed the fluorescent dyes by using the CD8 mouse anti-human antibody (R&D, MAB3801), FoxP3 rabbit anti-human antibody (Abcam, ab215206), GZMB rabbit anti-human antibody (Abcam, ab255598), IFNG mouse anti-human antibody (Abcam, ab218426), GFAP rabbit anti-human antibody (Abcam, ab68428), DAPI Staining Solution (Abcam, ab228549). Following the manufacturer’s instructions (Akoya, Opal Polaris 7 Color Automation IHC Detection Kit), we scanned the slides by using the Akoya Vectra Polaris Automated Quantitative Pathology Imaging System and quantified the results by using Akoya Inform software.

**Cell suspension preparation and flow cytometry**

Single-cell suspension was prepared via passing through a 40 μm cell strainer after cell harvest. Anti-CD16/32 (Biolegend) was incubated in recounted single-cell suspensions for 10 min at 4 °C, followed by fluorescent antibodies incubated staining (1:200) for 30 min at 4 °C. The following antibodies (Biolegend and BD) were used: Horizon™ Fixable Viability Stain 780, CD45 (30-F11), CD14 (M5E2), CD33 (P67.6), LILRB3 (MKT5.1). The population of M-MDSCs was identified by the surface marker expression (CD45+ CD14+ CD33+).

**Statistical Analysis**

Data were analyzed by R programming and commercially available software (SPSS 22.0). Categorically data was examined by chi-square, and the two-tailed t-test was for normally distributed variables. One-way analysis of variance (ANOVA) was used to assess the differences between groups. Risk factors for prognosis were examined by the COX model, and nonparametric data were examined by the Mann-Whitney U test. causal mediation analyses of survival outcomes based on Cox models. P < 0.05 was considered significant.

**DATA AVAILABILITY**

The available datasets analyzed in this study are listed in the following table and described in methods.

Table S1. Datasets analyzed in this study

| Datasets number | Download link |
| --- | --- |
| TCGA-glioma cohort | GDC Xena Hub, https://xenabrowser.net/datapages/ |
| CGGA-glioma cohort (China glioma genome atlas) | http://www.cgga.org.cn/ |
| GSE83300 | GEO, http://www.ncbi.nlm.nih.gov/geo/ |
| GSE68848 | GEO, http://www.ncbi.nlm.nih.gov/geo/ |
| Ivy GBM data | http://glioblastoma.alleninstitute.org/ |
| The Glioma Longitudinal AnalySiS (GLASS) | https://www.synapse.org/#!Synapse:syn17038081/wiki/585622 |
| Darmanis S *et al.* scRNA-seq data | http://gbmseq.org/ |
| Pombo Antunes *et al.* scRNA-seq data | http://www.brainimmuneatlas.org/ |
| 10x Spatial transcriptome data | https://github.com/theMILOlab/SPATAData |

**Study Approval**

The use of human tissues was approved (No. 2019-Y004) by the Ethics Committee of the School of Basic Medical Sciences, Fudan University, China. The use of human peripheral blood and 7-T MRI imaging data was approved by the Ethics Committee of the Huashan Hospital affiliated to Fudan University, China (KY2021-458, KY2020-275, respectively). All procedures were carried out according to the approved guidelines.

**AUTHOR contribution**

H.Y. and Y.M. designed and supervised this study. Q.Z. , H.W. and L.S. performed the bioinformatics analysis, and statistical analysis. Y.L. and anthor pathologists performed IHC staining, grading evaluation and cohort establishment. Z.L. and Q.Z. performed the IF staining and coculture. Q.Z., Y.L., H.W., H.Y., Y.M. prepared and revised the manuscript.

**SUPPLEMENTARY DISCUSSION**

Interactions between cancer cells and immune cells play important roles in modulating TME. However, the interactions between cellular states and specific biomarkers in immune cells remain poorly illustrated. In the present study, we demonstrated that the expression of LILRB3, which is mainly expressed on mo-TAMs, had a negative correlation with the overall survival of patients with all grades of gliomas. We utilized in vitro models and regression analyses to illustrate the correlations between LILRB3 expression in macrophages and the MES-like state of GBM. The findings indicated that tumor cellular state had an important influence on TIME. Combined with PD-1 expression, LILRB3 is a promising biomarker for identifying MES-like signatures of gliomas and immune-cold subtypes.

The chromosome 1p/19q co-deletion, initially described in 1994, is a strong independent favorable prognostic biomarker in both anaplastic and diffuse low-grade gliomas^10-12^. It was widely reported that glioma patients carrying 1p/19q codeletion had a longer survival and an improved radiotherapy response compared with those with 1p/19q non-codeletion status^13,14^. Notably, this robust biomarker has been used to classify gliomas into different subtypes^15,16^, and is considered as an essential complement to the grading system of 2021 WHO Classification of Tumors of the Central Nervous System^17^. Many genes located within the 1p/19q codeletion regions are potentially oncogenic^18^, of which several genes possess prognosis prediction value in stratified gliomas (e.g. FUBP1, CIC, etc.)^19^. However, the specific genes within these chromosomal regions associated with the immune status of TME remain largely unknown. The LILRB, genes are located in 19q13.42 and encode LIR receptors containing cytoplasmic ITIMs. We found that 19q13.42 was one of the most frequently deleted genomic loci in LGG. In addition, the expression of most genes located in 19q13.42 was significantly affected by 1p/19q co-deletion status. Notably, the expression of LILRB3 in bulk RNA-seq datasets might be contributed by both myeloid cells and cancer cells. Our scRNA-seq data revealed that LILRB3 was mainly expressed in myeloid cells (in which its expression was 10 times as high as that in neoplastic cells). Thus, the increase in LILRB3 expression with glioma grade indicates the accumulation of more myeloid cells in the tissues. Moreover, follow-up of patients involved in two large randomized control trials (RCTs) demonstrated that glioma patients with 1p/19q co-deletion benefited more from the combination of chemotherapy and radiotherapy^10,20^. These findings indicated that the expression of LILRB3 might have a predictive value for response to radiotherapy and chemotherapy. However, more studies are required to verify our conclusion.

Previous research reported that LILRB family proteins could bind to MHC class I molecules on antigen-presenting cells (APCs), subsequently transducing signals that inhibit immune response stimulation. Notably, the LILRB family proteins are also expressed in epithelial cells and cancer cells^21^, which might play different roles during environmental surveillance. Shiyu Ayukawa et al. reported that LILRB3 exhibited a high expression in epithelial cells and could interact with MHC class I to trigger a subsequent pathway, thereby generating mechanical force to remove precancerous cells^21^. Furthermore, low LILRB3 expression could deliver a stronger "eat me" signal to immune cells. Transformed cells with low LILRB3 expression were removed by natural killer cells or cytotoxic T cells^21^. In our study, a relatively low LILRB3 expression was also found in glioma patients with favorable prognosis (1p19q co-deletion). Therefore, the biological effects of 1p/19q co-deletion in gliomas might be associated with the expression of LILRB3.

Emerging evidence strengthens the perspective that genetic subtypes and cellular states of GBM are strongly associated with distinct TMEs, while signalings from TAMs can reciprocally modulate the tumor progression. Utilizing scRNA-seq and metabolism analyses, Mirco Friedrich et al. found that reorchestration of tryptophan metabolism in myeloid cells led to retardation of their differentiation^22^. A mesenchymal subtype of GBM is often correlated with mutations in the NF1 gene and can evoke significantly higher TME scores^8^. Moreover, Hara et al. identified crosstalks between macrophages and MES-like GBM cells. They found that oncostatin M (OSM) in macrophages could induce the transformation of GBM cells into the MES-like status via activation of the STAT3 signaling pathway^7^. Immunosuppressive macrophages could also impair the activation of T cells. Macrophages derived from PBMCs coculturing with MES-like GBM cells exhibited high expression of LILRB3, indicating that MES-like GBM cells could upregulate the expression of LILRB3 in TAMs. Notably, most GBM patients that responded to PD-1 inhibitors displayed lower MES-like scores in their lesions, whereas the non-responders generally had higher MES-like scores^7,23^. These observations indicate that high LILRB3 expression in TAMs combined with high PD-1 expression in T cells may contribute to a ‘cold’ TIME in GBM. Our study may inspire a new strategy that combines LILRB3-targeting therapy and immunotherapy for the treatment of GBM. In addition, we also demonstrate that LILRB3 is of significant prognostic value.

Immune archetypes defined by tumor gene expression or unique immune patterns have a profound influence on immunotherapy strategies. In gliomas, necrosis of neurons and other cells leads to a release of lipids and the formation of a high lipid environment within the tumor area, leading to a higher APOE expression in tumor core. APOE4 and APOE2 variants confer favorable and poor outcomes in melanoma, respectively, in contrast to their effects on Alzheimer’s disease^24^. Enhanced anti-tumor immune activation was observed in APOE4 mice relative to APOE2 mice, and T-cell depletion experiments demonstrated that the effect of APOE genotype on melanoma progression was mediated by altered anti-tumor immunity^24^. We observed increased binding of LILRB3 with APOE on the macrophage membrane following coculture with tumor cells. The relationship between APOE variants and immunotherapy response in glioma represents an intriguing area for future investigation.

Several biomarkers have been identified for the classification of TME, with PD-1/PD-L1 being among the most important. The expression level of PD-L1 has been reported to positively correlate with those of immunosuppressive molecules, including CTLA-4, TIM3, and LAG3^4^. These receptors mediate distinct immunosuppressive pathways, contributing to a ‘cold’ TME in gliomas. LILRB3 has been shown to inhibit the activity of cytotoxic T cells, which is critical for leukemia progression. LILRB3 expression has also been observed primarily on monocytic AML cells in FAB-M4 and -M5 subtypes of AML^25^. In this study, our single-cell RNA sequencing analysis revealed that LILRB3 was mainly expressed on clusters annotated as tumor-infiltrating monocytes and monocyte-derived TAMs. We also found that combining LILRB3 and PD-1 expression could classify glioma patients into distinct groups with varying numbers of TILs and cytotoxic T cells. Because the mechanism of LILRB3 for immunosuppression is different from PD-1/PD-L1, LILRB3 mainly targets the expression of myeloid cells and alters the microenvironment. It is urgent to develop a therapy antibody that targeting LILRB3 for further investigation of the effects of LILRB3 on immune suppression both in vitro and in vivo. These findings suggest that combining PD-1/PD-L1 inhibitors with receptor-targeting antibodies may achieve superior therapeutic efficacy compared to single-targeting strategies.

**SUPPLEMENTARY REFERENCE:**

1. Hu, Y., Hu, K., Song, H., Pawitan, Y., Piehl, F., and Fang, F. (2023). Infections among individuals with multiple sclerosis, Alzheimer's disease and Parkinson's disease. Brain Commun *5*, fcad065. 10.1093/braincomms/fcad065.

2. Feng, L., Qian, H., Yu, X., Liu, K., Xiao, T., Zhang, C., Kuang, M., Cheng, S., Li, X., Wan, J., and Zhang, K. (2017). Heterogeneity of tumor-infiltrating lymphocytes ascribed to local immune status rather than neoantigens by multi-omics analysis of glioblastoma multiforme. Sci Rep *7*, 6968. 10.1038/s41598-017-05538-z.

3. Madhavan, S., Zenklusen, J.C., Kotliarov, Y., Sahni, H., Fine, H.A., and Buetow, K. (2009). Rembrandt: Helping Personalized Medicine Become a Reality through Integrative Translational Research. Mol Cancer Res *7*, 157-167. 10.1158/1541-7786.Mcr-08-0435.

4. Chen, D., Li, G.P., Ji, C.X., Lu, Q.Q., Qi, Y., Tang, C., Xiong, J., Hu, J., Yasar, F.B.A., Zhang, Y., et al. (2020). Enhanced B7-H4 expression in gliomas with low PD-L1 expression identifies super-cold tumors. J Immunother Cancer *8*. ARTN e000154

10.1136/jitc-2019-000154.

5. Darmanis, S., Sloan, S.A., Croote, D., Mignardi, M., Chernikova, S., Samghababi, P., Zhang, Y., Neff, N., Kowarsky, M., Caneda, C., et al. (2017). Single-Cell RNA-Seq Analysis of Infiltrating Neoplastic Cells at the Migrating Front of Human Glioblastoma. Cell Rep *21*, 1399-1410. 10.1016/j.celrep.2017.10.030.

6. Pombo Antunes, A.R., Scheyltjens, I., Lodi, F., Messiaen, J., Antoranz, A., Duerinck, J., Kancheva, D., Martens, L., De Vlaminck, K., Van Hove, H., et al. (2021). Single-cell profiling of myeloid cells in glioblastoma across species and disease stage reveals macrophage competition and specialization. Nat Neurosci *24*, 595-610. 10.1038/s41593-020-00789-y.

7. Hara, T., Chanoch-Myers, R., Mathewson, N.D., Myskiw, C., Atta, L., Bussema, L., Eichhorn, S.W., Greenwald, A.C., Kinker, G.S., Rodman, C., et al. (2021). Interactions between cancer cells and immune cells drive transitions to mesenchymal-like states in glioblastoma. Cancer Cell *39*, 779-+. 10.1016/j.ccell.2021.05.002.

8. Neftel, C., Laffy, J., Filbin, M.G., Hara, T., Shore, M.E., Rahme, G.J., Richman, A.R., Silverbush, D., Shaw, M.L., Hebert, C.M., et al. (2019). An Integrative Model of Cellular States, Plasticity, and Genetics for Glioblastoma. Cell *178*, 835-+. 10.1016/j.cell.2019.06.024.

9. Zhuang, Q.Y., Li, F., Liu, J., Wang, H.Y., Tian, Y.C., Zhang, Z.G., Wang, F., Zhao, Z.H., Chen, J.C., and Wu, H.J. (2021). Nuclear exclusion of YAP exacerbates podocyte apoptosis and disease progression in Adriamycin-induced focal segmental glomerulosclerosis. Lab Invest *101*, 258-270. 10.1038/s41374-020-00503-3.

10. van den Bent, M.J., Carpentier, A.F., Brandes, A.A., Sanson, M., Taphoorn, M.J., Bernsen, H.J., Frenay, M., Tijssen, C.C., Grisold, W., Sipos, L., et al. (2006). Adjuvant procarbazine, lomustine, and vincristine improves progression-free survival but not overall survival in newly diagnosed anaplastic oligodendrogliomas and oligoastrocytomas: a randomized European Organisation for Research and Treatment of Cancer phase III trial. J Clin Oncol *24*, 2715-2722. 10.1200/JCO.2005.04.6078.

11. Cancer Genome Atlas Research, N., Brat, D.J., Verhaak, R.G., Aldape, K.D., Yung, W.K., Salama, S.R., Cooper, L.A., Rheinbay, E., Miller, C.R., Vitucci, M., et al. (2015). Comprehensive, Integrative Genomic Analysis of Diffuse Lower-Grade Gliomas. N Engl J Med *372*, 2481-2498. 10.1056/NEJMoa1402121.

12. Jenkins, R.B., Blair, H., Ballman, K.V., Giannini, C., Arusell, R.M., Law, M., Flynn, H., Passe, S., Felten, S., Brown, P.D., et al. (2006). A t(1;19)(q10;p10) mediates the combined deletions of 1p and 19q and predicts a better prognosis of patients with oligodendroglioma. Cancer Res *66*, 9852-9861. 10.1158/0008-5472.CAN-06-1796.

13. Iwadate, Y., Matsutani, T., Hasegawa, Y., Shinozaki, N., Higuchi, Y., and Saeki, N. (2011). Favorable long-term outcome of low-grade oligodendrogliomas irrespective of 1p/19q status when treated without radiotherapy. J Neurooncol *102*, 443-449. 10.1007/s11060-010-0340-4.

14. Chamberlain, M.C., and Born, D. (2015). Prognostic significance of relative 1p/19q codeletion in oligodendroglial tumors. J Neurooncol *125*, 249-251. 10.1007/s11060-015-1906-y.

15. Chan, A.K., Mao, Y., and Ng, H.K. (2016). TP53 and Histone H3.3 Mutations in Triple-Negative Lower-Grade Gliomas. N Engl J Med *375*, 2206-2208. 10.1056/NEJMc1610144.

16. Eckel-Passow, J.E., Lachance, D.H., Molinaro, A.M., Walsh, K.M., Decker, P.A., Sicotte, H., Pekmezci, M., Rice, T., Kosel, M.L., Smirnov, I.V., et al. (2015). Glioma Groups Based on 1p/19q, IDH, and TERT Promoter Mutations in Tumors. N Engl J Med *372*, 2499-2508. 10.1056/NEJMoa1407279.

17. Louis, D.N., Perry, A., Wesseling, P., Brat, D.J., Cree, I.A., Figarella-Branger, D., Hawkins, C., Ng, H.K., Pfister, S.M., Reifenberger, G., et al. (2021). The 2021 WHO Classification of Tumors of the Central Nervous System: a summary. Neuro Oncol *23*, 1231-1251. 10.1093/neuonc/noab106.

18. Chai, R.C., Zhang, K.N., Chang, Y.Z., Wu, F., Liu, Y.Q., Zhao, Z., Wang, K.Y., Chang, Y.H., Jiang, T., and Wang, Y.Z. (2019). Systematically characterize the clinical and biological significances of 1p19q genes in 1p/19q non-codeletion glioma. Carcinogenesis *40*, 1229-1239. 10.1093/carcin/bgz102.

19. Kamoun, A., Idbaih, A., Dehais, C., Elarouci, N., Carpentier, C., Letouze, E., Colin, C., Mokhtari, K., Jouvet, A., Uro-Coste, E., et al. (2016). Integrated multi-omics analysis of oligodendroglial tumours identifies three subgroups of 1p/19q co-deleted gliomas. Nat Commun *7*, 11263. 10.1038/ncomms11263.

20. Cairncross, G., Wang, M., Shaw, E., Jenkins, R., Brachman, D., Buckner, J., Fink, K., Souhami, L., Laperriere, N., Curran, W., and Mehta, M. (2013). Phase III trial of chemoradiotherapy for anaplastic oligodendroglioma: long-term results of RTOG 9402. J Clin Oncol *31*, 337-343. 10.1200/JCO.2012.43.2674.

21. Ayukawa, S., Kamoshita, N., Nakayama, J., Teramoto, R., Pishesha, N., Ohba, K., Sato, N., Kozawa, K., Abe, H., Semba, K., et al. (2021). Epithelial cells remove precancerous cells by cell competition via MHC class I-LILRB3 interaction. Nat Immunol *22*, 1391-1402. 10.1038/s41590-021-01045-6.

22. Friedrich, M., Sankowski, R., Bunse, L., Kilian, M., Green, E., Ramallo Guevara, C., Pusch, S., Poschet, G., Sanghvi, K., Hahn, M., et al. (2021). Tryptophan metabolism drives dynamic immunosuppressive myeloid states in IDH-mutant gliomas. Nat Cancer *2*, 723-740. 10.1038/s43018-021-00201-z.

23. Zhao, J., Chen, A.X., Gartrell, R.D., Silverman, A.M., Aparicio, L., Chu, T., Bordbar, D., Shan, D., Samanamud, J., Mahajan, A., et al. (2019). Immune and genomic correlates of response to anti-PD-1 immunotherapy in glioblastoma. Nat Med *25*, 462-469. 10.1038/s41591-019-0349-y.

24. Ostendorf, B.N., Bilanovic, J., Adaku, N., Tafreshian, K.N., Tavora, B., Vaughan, R.D., and Tavazoie, S.F. (2020). Common germline variants of the human APOE gene modulate melanoma progression and survival. Nat Med *26*, 1048-1053. 10.1038/s41591-020-0879-3.

25. Wu, G., Xu, Y., Schultz, R.D., Chen, H., Xie, J., Deng, M., Liu, X., Gui, X., John, S., Lu, Z., et al. (2021). LILRB3 supports acute myeloid leukemia development and regulates T-cell antitumor immune responses through the TRAF2-cFLIP-NF-kappaB signaling axis. Nat Cancer *2*, 1170-1184. 10.1038/s43018-021-00262-0.

**SUPPLEMENTARY UNCROPPED WB (corresponding to Figure 2H):**


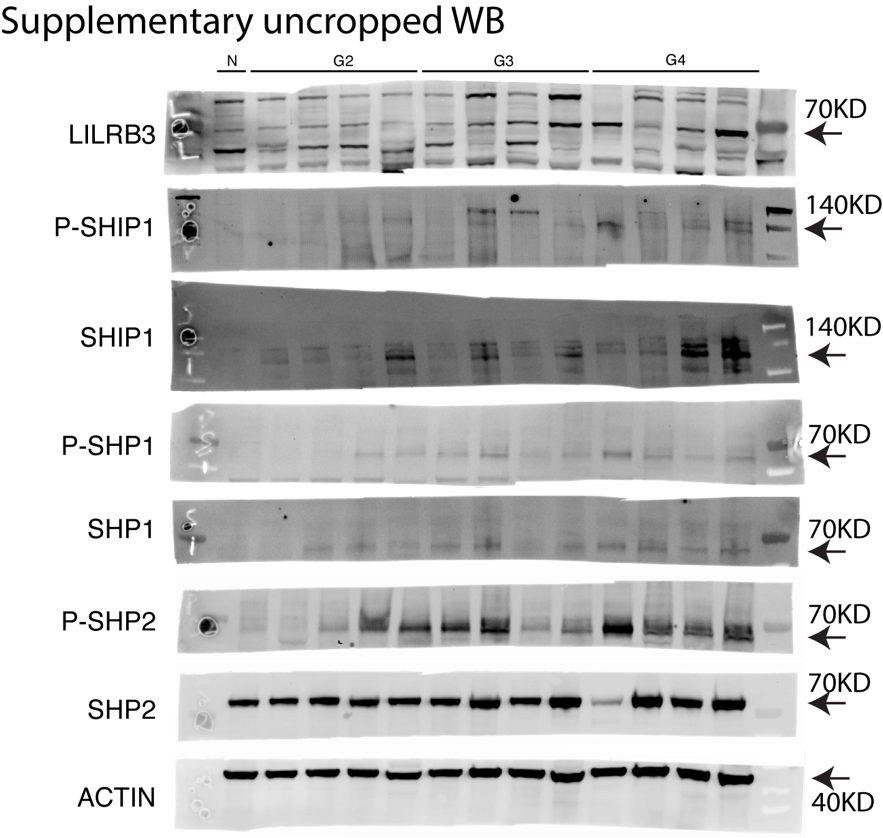

Supplement: Supplementary file 11 — Supporting Information [file CTM2-13-e1396-s008.docx]
